# Overexpression of miR-20a-5p in Tumor Epithelium Is an Independent Negative Prognostic Indicator in Prostate Cancer—A Multi-Institutional Study

**DOI:** 10.3390/cancers13164096

**Published:** 2021-08-14

**Authors:** Maria J. Stoen, Sigve Andersen, Mehrdad Rakaee, Mona I. Pedersen, Lise M. Ingebriktsen, Tom Donnem, Ana P. G. Lombardi, Thomas K. Kilvaer, Lill-Tove R. Busund, Elin Richardsen

**Affiliations:** 1 Translational Cancer Research Group, Institute of Medical Biology, UiT The Arctic University of Norway, N-9037 Tromso, Norway; limain@online.no (L.M.I.); ana.lombardi@uit.no (A.P.G.L.); thomas.k.kilvar@uit.no (T.K.K.); lill-tove.rasmussen.busund@unn.no (L.-T.R.B.); elin-ri@live.no (E.R.); 2 Translational Cancer Research Group, Institute of Clinical Medicine, UiT The Arctic University of Norway, N-9037 Tromso, Norway; sigve.andersen@uit.no (S.A.); mehrdad.rakaee@uit.no (M.R.); mona.i.pedersen@uit.no (M.I.P.); tom.donnem@uit.no (T.D.); 3 Department of Oncology, University Hospital of North Norway, N-9038 Tromso, Norway; 4 Department of Medicine, Brigham and Women’s Hospital, Harvard Medical School, Boston, MA 02115, USA; 5 Centre for Cancer Biomarkers CCBIO, Department of Clinical Medicine, Section for Pathology, University of Bergen, N-5021 Bergen, Norway; 6 Department of Clinical Pathology, University Hospital of North Norway, N-9038 Tromso, Norway

**Keywords:** prostate cancer, microRNA, miR-20a-5p, radical prostatectomy, biomarker, prognostication

## Abstract

**Simple Summary:**

MicroRNAs (miRs) have critical regulatory roles in cell functions, and are involved in prostate cancer tumorigenesis. miR-20a-5p is a member of the oncogenic miR-17-92 cluster. Overexpressed miR-20a-5p has been shown to increase both cell proliferation and cell migration in cancers. The aim of our cohort study was to evaluate the prognostic role of miR-20a-5p in prostate cancer. We found miR-20a-5p associated with biochemical failure in tumor epithelium and tumor stroma. In the multivariable analysis miR-20a-5p in tumor epithelium was found to be an independent prognostic predictor for biochemical failure. In the functional studies, migration and invasion were significantly increased in miR-20a-5p transfected prostate cancer cell lines. In conclusion, high miR-20a-5p expression in tumor epithelium is a negative independent prognostic factor for biochemical failure in prostate cancer.

**Abstract:**

Objective: assessing the prognostic role of miR-20a-5p, in terms of clinical outcome, in a large multi-institutional cohort study. Methods: Tissue microarrays from 535 patients’ prostatectomy specimens were constructed. In situ hybridization was performed to assess the expression level of miR-20a-5p in different tissue subregions: tumor stroma (TS) and tumor epithelium (TE). In vitro analysis was performed on prostate cancer cell lines. Results: A high miR-20a-5p expression was found negatively in association with biochemical failure in TE, TS and TE + TS (*p* = 0.001, *p* = 0.003 and *p* = 0.001, respectively). Multivariable analysis confirmed that high miR-20a-5p expression in TE independently predicts dismal prognosis for biochemical failure (HR = 1.56, 95% CI: 1.10–2.21, *p* = 0.014). Both DU145 and PC3 cells exhibited increased migration ability after transient overexpression of miR-20a-5p, as well as significant elevation of invasion in DU145 cells. Conclusion: A high miR-20a-5p expression in tumor epithelium is an independent negative predictor for biochemical prostate cancer recurrence.

## 1. Introduction

Prostate cancer (PCa), with an estimated 1.3 million new cases in 2018, is the second most common cancer diagnosis among men worldwide [1]. PCa was responsible for ~360,000 deaths in 2018, and is thereby the fifth most common cause of cancer death in men [1]. PCa varies from indolent to aggressive cancer, and with increased diagnostics comes overtreatment, with its associated side effects. Current treatment stratification is hampered by imprecise large risk groups [2]. Considering that the only established biomarker, serum Prostate Specific Antigen (PSA) has low specificity [3], new PCa biomarkers that can more precisely distinguish cancer aggressiveness are highly warranted [4].

MicroRNAs (miRs) are small, non-coding, single stranded RNA molecules about 22 nucleotides in length [5]. They bind complementary to the 3′-untranslated region (3′ UTR) of the target gene, which causes mRNA degradation or inhibition of translation [6]. miRs are dysregulated in various cancers [4], and can function as oncomirs or tumor suppressor miRs determined by the target transcripts [7]. They are involved in mechanisms driving cancer aggressiveness, such as cell proliferation, migration, invasion, apoptosis, angiogenesis and radio- and chemoresistance [7]. Dysregulated miRs in circulation are associated with tumor growth, drug resistance and metastasis, making them potential diagnostic and prognostic biomarkers for different cancers [3], including PCa [4,8].

The miR-17-92 cluster, located in an intron of MIR17HG (C13orf25) on chromosome 13, comprises six members: miR-17, miR-18a, miR-19a, miR-19b, miR-20a and miR-92a [9]. The cluster is important for normal development, and contributes to regulating cellular processes such as proliferation, cell cycle progression and apoptosis [10]. The miR-17-92 cluster is often dysregulated in solid and hematopoietic malignancies, hence it is called ‘oncomiR-1′ [11].

High expression of miR-20a is shown to increase cell proliferation and induce migration in different cancers such as gastric, lung and hepatocellular carcinoma [12,13,14]. High miR-20a expression is also correlated with high Gleason score (≥7), which is the strongest prognostic marker in early stage prostate cancer [15]. Basic studies show that miR-20a targets the E2F family, p21 and p57, thus blocking cell cycle checkpoints and promoting tumorigenesis [10,16]. High miR-20a-5p expression has been associated with biochemical failure (BF) and tumor recurrence after radical prostatectomy, and consequently suggested as a potential prognostic biomarker in PCa [17]. However, Ottman and colleagues found a tumor suppressor effect of the whole miR-17-92 cluster [18].

Using microarray expression analysis (no. of assessed miRs: 1435) in a subset of patients (*n* = 30) of current cohort, we have previously found miR-20a-5p to be significantly upregulated in PCa patients with a rapid BF vs. non-BF [19]. Since the prognostic impact of miR-20a-5p has not been thoroughly evaluated in the whole cohort, we sought to evaluate in situ expression of miR-20a-5p in human PCa tissue. Our second aim was to perform in vitro analyses to study the effects of miR-20a-5p on proliferation, migration and invasion, and thereby validate the in situ results.

## 2. Materials and Methods

### 2.1. Patients

We retrospectively identified 671 consecutive patients who received radical prostatectomies for adenocarcinoma of the prostate gland between 1995 and 2005, from the archives of the departments of Pathology from three Norwegian hospitals: Trondheim University Hospital (St. Olav, *n* = 341); Nordlandssykehuset Bodø (NLSH, *n* = 63); and the University Hospital of North Norway (UNN, *n* = 267). Of the 671 patients, 136 were excluded, thus leaving 535 patients with available tissue and sufficient follow-up data. Patients were excluded due to inadequate tissue blocks (*n* = 130), other types of cancer within 5 years of PCa diagnosis (*n* = 4), missing follow-up data (*n* = 1) and pelvic radiotherapy prior surgery (*n* = 1). None received pre-operative hormonal therapy. Relevant clinical data was registered and outcome data was collected until the last follow-up (31 December 2015). The median follow-up was 150 months (18–240 months).

BF was defined as a PSA ≥ 0.4 ng/mL in two consecutive postoperative blood samples [20]. Clinical failure (CF) was defined as clinically palpable recurring tumor or metastasis to lymph nodes, visceral organs or bones verified by radiology. Lastly, prostate cancer death (PCD) was defined as PCa as death-cause stated in the patient’s journal. BF-free survival (BFFS), CF-free survival (CFFS) and PCD-free survival (PCDFS) was calculated from time of surgery to either the last follow-up date or date at which BF, CF or PCD occurs. An experienced uro-pathologist (ER) re-evaluated the tumor-samples in 2018, and graded them according to the WHO 2016 guidelines [21,22]. Cancer of the Prostate Risk Assessment Postsurgical Score (CAPRA-S Score) was used to predict outcomes after radical prostatectomy. This variable was calculated based on ISUP Grade group, PSA value, margin, extracapsular extension, lymph node invasion and seminal vesicle invasion [23]. The REMARK guidelines were followed in regards to the reporting of biomarker expression and clinicopathological variables, and the analysis of survival data [24]. See our previously published report for more detailed information regarding the cohort [25].

### 2.2. Tissues and Tissue Microarray Construction (TMAs)

The most representative areas of TE and adjacent stromal areas were demarked for sampling by an experienced uro-pathologist (ER). The TMAs were made by using a tissue-arraying instrument (Beecher Instruments, Silver Springs, MD, USA), which harvested cores with 0.6 mm in diameter. The cores were inserted into paraffin blocks, and 4 μm sections were cut by a Micron microtome (HM355S). The detailed methodology has previously been reported [26].

### 2.3. In Situ Hybridization (ISH)

**Figure 1:**
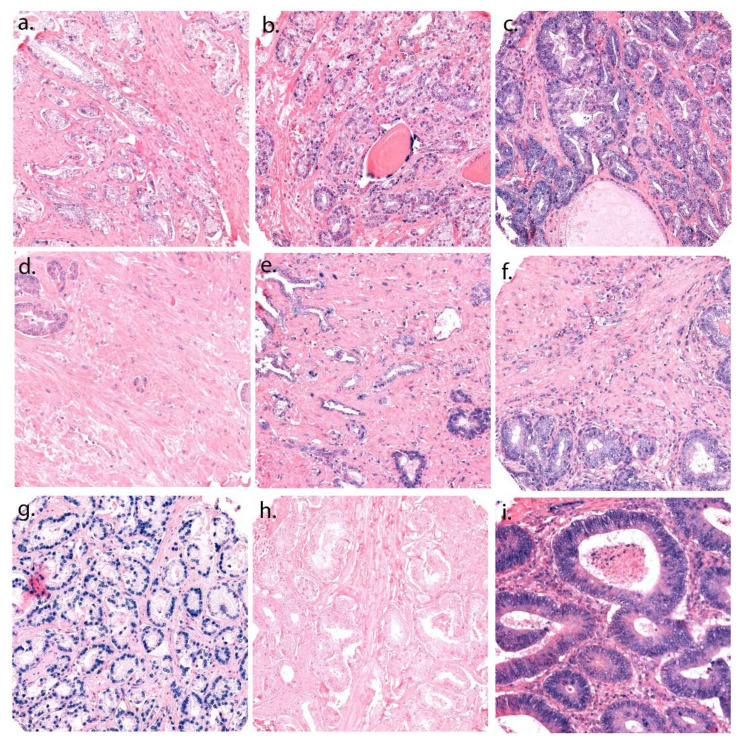
A panel of ISH stained cores; representative scorings of miR-20a-5p in tumor epithelium (TE) and tumor stroma (TS). TE is given a value from 0 to 3, depending on the intensity of the staining in both core and cytoplasm of the TE. TS is given a value from 0 to 3, depending on the density of positive TS cells in the examined core. (**a**–**c**) Score 1–3 in TE; (**d**–**f**) Score 1–3 in TS; (**g**) U6 control staining; (**h**) Scramble miR; (**i**) Positive tissue control: normal human colon tissue, 20× magnification.

The Ventana Discovery Ultra instrument (Ventana Medical Inc., Marana, AZ, USA) performed the chromogenic ISH, with buffers and detection reagents purchased from Roche (Basel, Switzerland). All miRCURY LNA detection probes; hsa-miR-20a-5p, (No. 611011-360), neg. control (scrambled-miRNA, No. 157057117), and pos. control (U6 hsa, No. 160010126), were from Exiqon (Vedbaek, Denmark). To optimize the detection method, unmasking pretreatments and probe concentrations were tested on a TMA multiorgan block. To confirm the staining, miR-20a-5p expression in malignant and healthy tissue was studied on control TMA blocks. To stop RNA degradation, RNAse-free water, buffers and equipment were utilized. Recommended temperatures were guidelines for optimizing hybridization temperatures for the probes and controls. To achieve optimal concentrations of each miR-probe, tests at different concentrations were conducted and clear staining without unspecific positive background staining were chosen. The detection-probe supplier (Exiqon) recommend miR-probe concentrations in the range of 20–80 nM. To guarantee the sensitivity level of the ISH method a U6 snRNA control probe (1.5 nM concentration) was utilized. For U6, the best sensitivity was considered a bright nuclear signal at concentrations between 0.1–2.0 nM. A strong nuclear staining visualized by a light microscope implies low amounts of RNA degradations in U6. For the scrambled-miRNA (negative control), the concentration was 10 nM, and 50 nM for miR-20a-5p. Our protocol steps for the ISH procedure are documented in our previous study [27]. The temperatures for the different probes were 40 °C (miR-20a-5p), 55 °C (U6) and 57 °C (scramble miR) for the hybridization, and the incubation time was 60 min. Figure 1 contains the staining controls.

### 2.4. Cell Culture

We used two different PCa cell lines to evaluate the functional properties of miR-20a-5p. These were the human androgen-independent PCa cell lines PC3 (ATCC CRL-1435) derived from bone metastasis and DU145 (ATCC HTB-81) derived from brain metastasis. In every experiment passage below 46 and 69 was used for PC3 and DU145, respectively. Opti-MEM I (1x) medium without phenol red (cat.# 11058-021, GIBCO, RF, UK) was used for the cultured cells (2 × 10^5^ cells/mL), and supplemented with Penicillin Streptomycin 1% (cat.# 15140-148, Gibco, NY, USA) and 5% of fetal bovine serum (cat.# S0415, Biochrom, Berlin, Germany), in a humidified atmosphere with 5% CO_2_: 95% air, at 37 °C, for 72 h. Then, we replaced the culture medium with one without serum 24 h before the experiments, where the cells were 85–90% confluent.

### 2.5. Viability Assay

3 × 10^3^ cells/well (PC3) Additionally, 5 × 10^3^ cells/well (DU145) were cultured in 96-well plates for the colorimetric proliferation assay. Cells were, at different time points, incubated with 12 mM of [3-(4,5-dimethylthiazol-2-yl)-2,5-diphenyltetrazolium bromide] (MTT, 5 mg/mL) (cat.# M6494, Invitrogen, OR, USA). The formazan crystals produced were solubilized by addition of 0,01 M HCl/SDS (cat.# 28312, Thermo Scientific, Chicago, IL, USA) for 4 h at 37 °C, and mixed thoroughly with the pipette. In order to dissolve the formazan, the cells were incubated at 37 °C overnight. The absorbance was measured at 570 nm, in the CLARIOstar plate reader (BMG Labtech, Ortenberg, Germany).

### 2.6. Cell Transfection

We used 10 µM has-miR-20a-5p Pre-miR miRNA Precursor (catalog# AM17100, Thermo Fisher Scientific, Scientific instrumentation, reagents and consumables, and software services, Waltham, MA, USA), and Cy3 Dye-Labeled Pre-miR Negative Control #1 (catalog# AM17120, Thermo Fisher Scientific, USA) to transiently transfect the cells. For this, the transfection reagent Lipofectamine RNAiMAX (catalog#13778075, Thermo Fisher Scientific, USA) was utilized. If exposed to UV-light, the transfected Cy3 Dye-Labeled Pre-miR Negative Control emits fluorescent light. The transfection efficiency was, by fluorescence microscope, evaluated to be at 80–95%.

### 2.7. Wound Healing Analysis

The wound healing assays were performed with miR-20a-5p transfection in PC3 and DU145 PCa cell lines. For the analysis, 2 × 10^5^ cells/well were grown in a 24-well plate. PBS was used to wash the PC3 and DU145 cell lines. The cells were then incubated in serum free culture medium containing a blocking DNA replication mitomycin C (10 µg/L) to avoid cell proliferation. We used 200 µL sterile pipette tips to wound the cells. Then, the cells were washed to remove detached cells and debris [28], and after 4 h they were transfected with has-miR-17-5p Pre-miR miRNA Precursor (catalog# PM12412, Thermo Fisher Scientific, USA) or Cy3 Dye-Labeled Pre-miR Negative Control #1 (catalog# AM17120, Thermo Fisher Scientific, USA) (control, basal level of cellular function) for 24 h at 37 °C. The same area of the wound was photographed at 0 and 24 h in order to measure wound-closure in cells transfected with miR-20a-5p and in controls [28]. Nikon Eclipse TS100 inverted optical microscope was used to capture the images, and Micrometrics SE Premium 4 software was used for analyzation. The background levels at 0 h were subtracted, and after 24 h of incubation the areas occupied by migrating cells (transfected and control cells) was calculated. The results were plotted (mean ± SEM) in relation to the control (C = 1).

### 2.8. Invasion Assays

2 × 10^5^ cells (PC3 and DU145 cell line) in serum free culture medium were seeded in ThincertR chambers (Greiner Bio-one, Kremsmünster, Austria) with polyethylene terephthalate membranes (pore size = 8 mm) pre-coated with 50 μL of phenol red-free Matrigel (BD, Gibco). We used 24-well plates for placing the chambers, and they contained culture medium with 5% FBS in the lower chamber [28,29]. DU145 and PC3 cells in upper chambers were incubated with Cy3 Dye-Labeled Pre-miR Negative Control #1 (catalog# AM17120, Thermo Fisher Scientific, USA) (control, basal level of cellular function) or has-miR-20a-5p Pre-miR miRNA Precursor (catalog# AM17100, Thermo Fisher Scientific, USA) (10 μM) at 37 °C for 48 h. Protocols for cell invasion analyses have previously been published [28]. PBS (10 mM) was used to wash the chambers, then fixed for 30 min in paraformaldehyde (4%), and stained for 10 min with crystal violet (0.2%). Non-invading cells from the membrane upper surface were removed by a cotton swab. Membranes containing invaded cells under the membrane surface were photographed, and by using Nikon Eclipse TS100 (inverted optical microscope) images were captured in duplicate of three random microscope fields. Image J software was utilized to determine the area of cell invasion, and the results were plotted (mean ± SEM) in relation to control (C = 1).

### 2.9. Statistical Methods

All statistical analyses were conducted with the SPSS software, version 26 (IBM, SPSS Inc., Chicago, IL, USA). Correlations between miR-20a-5p expression and clinicopathological variables were explored by Pearson’s Chi-square analysis and Fisher’s exact test. The Kaplan–Meier method was utilized to draw univariate survival curves. Presentations of the curves were terminated when under 10% of patients were at risk (at 192 months). The log-rank test assessed the statistical significance between survival curves. Cox regression analysis (backward conditional) was used for the multivariate analysis, with a probability for stepwise entry and removal at 0.05 and 0.10, respectively. Variables significant from the univariate analysis were included in the multivariate analysis. *p*-values < 0.05 was deemed significant for all analyses. A two-way random effect model with absolute agreement was used to test interobserver reliability between the two scorers.

## 3. Results

### 3.1. Patient Characteristics

The last follow-up data for this study were from 31 December 2015. By then, 37% (*n* = 200), 11% (*n* = 56) and 3.4% (*n* = 18) of the patients experienced BF, CF or PCD, respectively. Median age at surgery was 62 years (47–75 years), median serum PSA level was 8.8 ng/mL (range 0.7–104 ng/mL), and median tumor size was 20 mm (2–50 mm). pT-stage included T2, T3a and T3b (*n* = 374, 114 and 47, respectively), while ISUP Grade Group ranged from 1–5 (*n* = 183, 220, 80, 19 and 33, respectively). Detailed information regarding clinicopathological characteristics for the 535 patients included in the study have been previously published [25].

### 3.2. Scoring of miR-20a-5p Expression and Cut-Off Values

Figure 1 demonstrates representative scores for miR-20a-5p expression in TE and TS. An experienced uro-pathologist (ER) and a trained investigator (LMI/MJS) scored the TMA cores semi-quantitatively, and blinded to each other’s scores and outcome. The scoring model is based on an overall nuclei and cytoplasmic staining intensity. The average staining intensity in TE is given a value from 0 to 3 (0 = negative, 1 = weak, 2 = moderate and 3 = strong). In TS, the density of positive cells was scored (0 = 0%, 1 = 1–20%, 2 = 21–50% and 3 > 50%). Predefined median cut-off values were used for the dichotomization of the variables. A high miR-20a-5p expression was defined as a score ≥ 2 for TE and ≥1.5 for TS. By adding dichotomized values from the TE- and the TS-variables together, we created the TE + TS variable. The TE + TS variable has three categories; low TE/TS (0/0), mixed (0/1 or 1/0), and high TE/TS (1/1). Of the patients, 406 had valid scores, while 129 were categorized as missing, considering they had no malignant cells in their cores, or the patient’s cores had fallen off the slide during staining. An excellent scoring agreement was achieved between the uro-pathologist (ER) and the trained investigators (LMI/MJS). The intraclass correlation coefficient was 0.93 (*p* < 0.001) for miR-20a-5p in TE and 0.79 (*p* < 0.001) in TS.

### 3.3. Proliferation, Migration and Invasion Assays

#### 3.3.1. Proliferation

**Figure 2:**
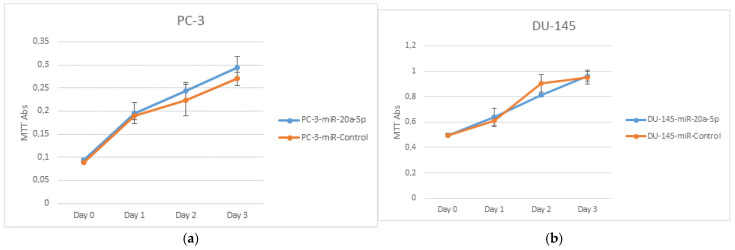
PC3 (**a**) and DU145 (**b**) cell lines were used to assess cell proliferation. PC3 and DU145 transfected with miR-20a-5p were compared to control cells. There were no significant differences in proliferation in PC3 or DU145 cell lines.

DU145 and PC3 cell lines were transfected with miR-20a-5p (10 μM) after being plated for 24 h. The cells were treated with MTT over 4 days. No significant difference in proliferation was observed in the cells overexpressing miR-20a-5p compared to controls (non-transfected DU145 and PC3, Figure 2).

#### 3.3.2. Migration

**Figure 3:**
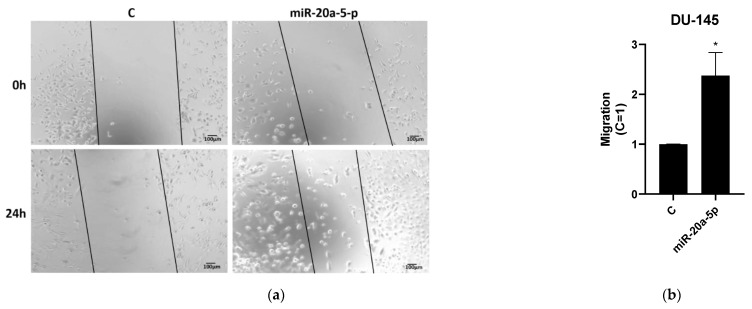

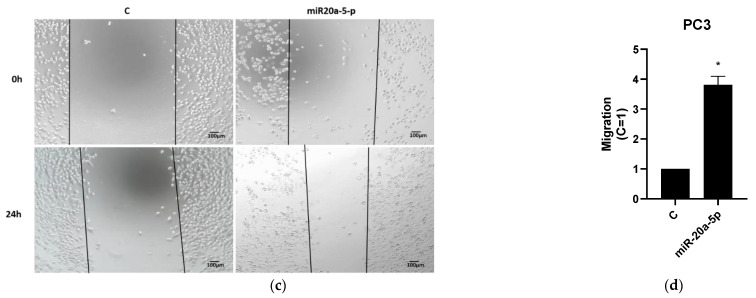
Wound healing analysis of transfected miR-20a-5p cells compared to controls in PC3 and DU145 cell lines. PC3 and DU145 cell lines showed significant (*p* < 0.05) migration compared to controls. Results are plotted (mean ± SEM) in relation to controls (C = 1). Figure 3 shows representative results of three experiments, performed in duplicates, while complete results from all experiments are presented in Supplementary Table S1. (**a**,**b**) Wound healing assay for DU145 cell lines; (**c**,**d**) Wound healing assay for PC3 cell lines. * Significantly different from control (*p* < 0.05, Student *t*-test).

In the wound healing assays, DU145 and PC3 cell lines were transfected with miR-20a-5p to study the effect of miR-20a-5p on cell migration. There was a significant increase in migration at 24 h for miR-20a-5p transfected cells for PC3 (*p* < 0.05) and DU145 (*p* < 0.05) cell lines compared to controls, using Student’s *t*-test. Figure 3 shows representative results of three different experiments for both PC3 and DU145 cell lines.

#### 3.3.3. Invasion

**Figure 4:**
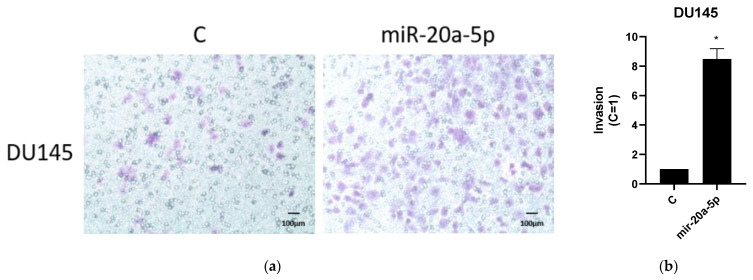

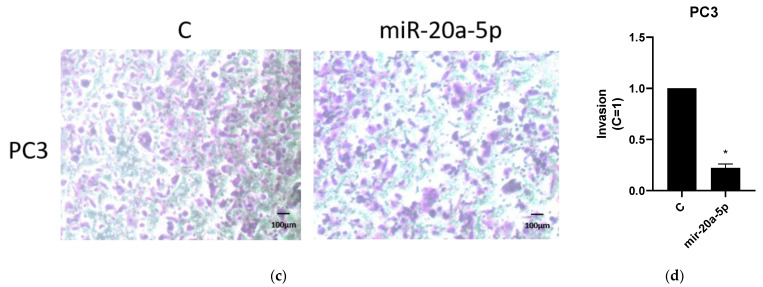
Invasion analysis of transfected miR-20a-5p cells compared to controls in PC3 and DU145 cell lines (representative of three experiments). DU145 cell lines showed significant (*p* < 0.05) migration compared to controls. Results are plotted (mean ± SEM) in relation to controls (C = 1). Complete results from all experiments are presented in Supplementary Table S2. (**a**,**b**) Invasion assay for DU145 cell lines; (**c**,**d**) Invasion assay for PC3 cell lines. * Significantly different from control (*p* < 0.05, Student *t*-test).

Invasion analysis was used to study the effects of transfected miR-20a-5p on invasion of DU145 and PC3 PCa cell lines. Representative results from three experiments of invasion assays for DU145 and PC3 are presented in Figure 4. After miR-20a-5p treatment, the invasion had significantly increased in DU145 cell lines (*p* < 0.05), by Student’s *t*-test.

### 3.4. miR-20a-5p Correlations

Perineural infiltration correlated with high miR-20a-5p expression in TE, TS and TE + TS (*p* = 0.001, *p* = 0.001 and *p* = 0.001, respectively). Positive apical margin (PAM) correlated with high miR-20a-5p expression in TE, TS and TE + TS (*p* = 0.007, *p* = 0.037 and *p* = 0.018, respectively).

### 3.5. Univariate Analysis

**Figure 5:**
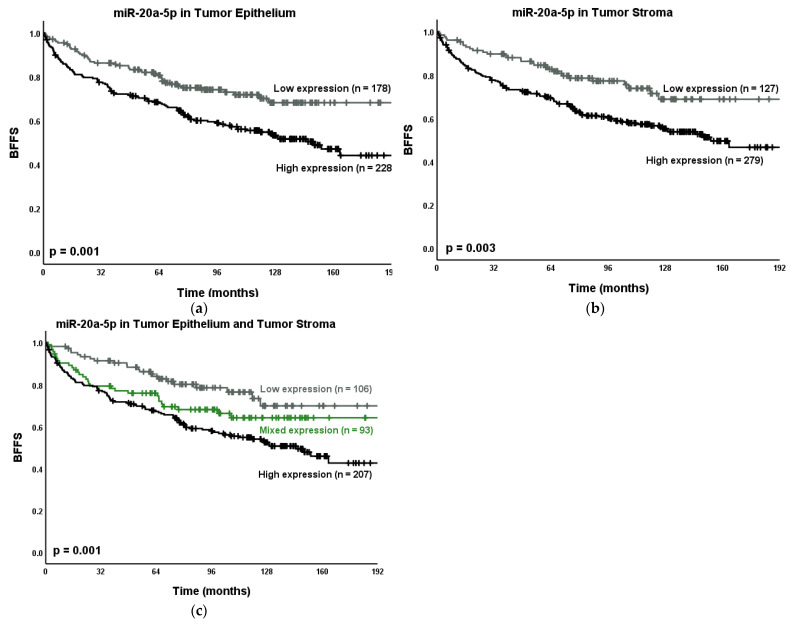
Kaplan–Meier curves presenting the relations between the PCa outcome BF and miR-20a-5p expression in (**a**) TE; (**b**) TS and (**c**) TE + TS. Abbreviations: BFFS = Biochemical failure-free survival; CFFS = clinical failure-free survival; *n* = number; *p* = *p*-value.

The results from the univariate analysis for miR-20a-5p and BF, CF and PCD are presented in Table 1, Figure 5, and Supplementary Figure S1. The results for univariate analyses for clinicopathological variables are available in the supplementary file (Supplementary Table S3). High miR-20a-5p expression was significantly associated with BF (TE: *p* = 0.001, TS: *p* = 0.003, TE + TS: *p* = 0.001). We did not find significant associations between miR-20a-5p and CF or PCD. However, there is not enough statistical power to properly assess these associations.

### 3.6. Multivariate Analysis

The results from the multivariate analyses for BF and miR-20a-5p are presented in Table 2. In multivariate analyses, high miR-20a-5p expression in TE (HR = 1.56, 95% CI: 1.10–2.21, *p* = 0.014) and TE + TS high/high expression (HR = 1.75, 95% CI: 1.10–2.78, *p* = 0.018) were independently associated with BF. The clinicopathological variables significant for BF were tumor size, CAPRA-S and perineural infiltration.

## 4. Discussion

In our study, we found high miR-20a-5p expressions in TE and TE + TS to be independent negative prognostic factors for BF. To our knowledge, this is the first prognostic study using ISH to examine miR-20a-5p expression in PCa tissue. The strengths of our study include a long follow-up time (median 12.5 years), a large and unselected patient population (*n* = 535) and the possibility of evaluating miR-20a-5p expression in both TE and TS. High miR-20a-5p expression can also be analyzed by ISH on routine paraffin-embedded tissue. The study’s limitations comprise the retrospective study design, the low incidences of CF and PCD (56 and 18, respectively) and the lack of healthy tissue controls.

Our results are supported by previous studies using real-time reverse transcription PCR (RT-PCR) to evaluate the prognostic impact of miR-20a-5p in PCa [17,30,31]. Qiang et al. found a shorter mean survival time in patients with a high expression of miR-20a in their PCa tissue samples [30]. By studying serum collected from radical prostatectomy patients, Hoey et al. discovered that a high expression of miR-20a-5p was associated with a high Gleason score, and a predictor for high-risk disease [17]. Moreover, they found an association between high miR-20a-5p expression and a shorter BFFS in the Cancer Genome Atlas (TCGA) dataset [17]. Lin et al. studied blood samples from 97 castration-resistant PCa patients, and found an association between shorter survival time and a high miR-20a expression before docetaxel treatment [31]. In addition, having an unchanged or decreased miR-20a expression post docetaxel treatment was an independent predictor for worse overall survival (OS) shown by Cox regression analysis [31]. To our knowledge, our study is the largest study evaluating miR-20a-5p expression and the endpoints BF, CF and PCD in PCa patients.

Several studies have explored the association between miR-20a-5p expression and clinicopathological variables. Shen et al. found miR-20a to be upregulated in plasma from patients with pT-stage 3 compared to stage 2 and 1, as well as in patients with a high risk (CAPRA score) [32]. Moreover, they reported that miR-20a in combination with three other miRs can distinguish low vs. high risk PCa patients using the D’Amico scores [32]. Other studies have reported significantly higher levels of miR-20a in PCa tissue from patients with a high Gleason score (score > 6), compared to low Gleason scores (score ≤ 6) [15,30]. miR-20a-5p was one of four miRs to have high connectivity with important genes according to an interaction network, and might therefore be important for PCa development [33]. In our study, we found significant correlations between miR-20a-5p and perineural infiltration and positive apical margin.

Zhang et al. performed a systematic review where they evaluated the prognostic implication of miR-20a in PCa [34]. In this article, miR-20a was found to be upregulated in the circulatory system in PCa, and this was correlated with a worse OS [34]. They also found miR-20a upregulated in gastric cancer, glioblastoma, lymphoma and non-small-cell lung cancer (NSCLC), correlating with a worse OS and DFS [34]. Some systematic reviews have also suggested circulating miR-20a as a diagnostic biomarker for NSCLC [35,36]. Meta-analyses focusing on gastrointestinal cancers have found high miR-20a expression (tissue and circulation) associated with poor prognosis [34,37,38,39,40]. In nasopharyngeal cancer, a systematic review found upregulated miR-20a in the circulation associated with poor prognosis [41]. These meta-analyses and systematic reviews imply the same results in patient outcome as our study.

miR-20a-5p is a part of the miR-17-92 cluster, which has been considered to have an oncogenic function in PCa [42,43], although Ottman et al. disagreed [18]. Many studies have measured an upregulation of miR-20a in PCa tissue [30,44,45,46,47] and in serum from PCa patients before radical prostatectomy compared to healthy controls [48].

Our study used wound healing assays to analyze the effects of miR-20a-5p on migration, and the results were significant migration in both PC3 and DU145 cell lines. We also found significant results for miR-20a-5p transfection on invasion in DU145 cell lines. Several in vitro studies have described similar functional aspects of miR-20a in PCa cell lines [17,30,43,45]. Qiang et al. reported that miR-20a induces cell invasion and migration in PC3 and DU145 cells [30]. Hoey et al. found that miR-20a-5p overexpression increased colony formation in a 3D agar matrix and lead to PC3 cell survival after radiation treatment [17]. The increased migration and invasion after miR-20a induction have been explained through a few different cellular mechanisms. Liu et al. found that SOX4 upregulates miR-20a in PCa, and overexpression of this gene is associated with a poor prognosis, and causes cell proliferation, migration and invasion in PCa cells [43]. Qiang et al. proposed that miR-20a contributes to PCa progression by inhibiting the non-receptor tyrosine kinase (ABL2) and thereby promoting cell invasion and migration in PCa cells in vitro [30]. Lastly, high miR-20a inhibits the gap junction protein CX43, contributing to the proliferation of PCa cells in vitro [45]. In this study, we found that overexpressed miR-20a-5p increased invasion in DU145 cell lines, but decreased invasion in PC3 cell lines. Considering DU145 cell lines are derived from brain metastasis and PC3 from bone metastasis, these cells express different proteins and miR-levels. There is a need for more cell studies in order to understand miR’s roles in the signaling pathways involved in invasion for PC3 and DU145.

In our study we performed proliferation studies in PC3 and DU145 cell lines, but found no significant difference compared to controls. We hypothesize that this result might be due to the fact that cell line studies have several limitations. Some cell lines unfortunately show only marginal similarity to original primary phenotype, they can lack polarity and morphological features and they can have altered genomic content. Cell line studies have shown miR-20a to enhance proliferation in MDA-PCa-2b cells [45], decrease apoptosis in PC3 cells [16] and cause tumor growth in vivo [30,45]. The oncogenic activity of miR-20a has been seen through different mechanisms. For example, Sylvestre et al. found that translation of the E2F family of transcription factors is regulated by miR-20a, which are important for cell cycle regulation and apoptosis [16]. Other studies found miR-20a to inhibit the tumor suppressors RB1 and PTEN in DU145 cell lines [43,49].

Our study adds to the growing literature on the oncogenic activity of miR-20a in PCa. Other studies describe upregulated miR-20a measured in serum from PCa-patients [17,31,34,48]. However, our study implies miR-20a-5p expression in prostatectomy specimens has prognostic value on PCa together with an established risk score.

## 5. Conclusions

In this study, we found high miR-20a-5p expression in TE and in TE + TS to be a negative independent prognostic factors for BF. We also found significantly higher migration in miR-20a-5p transfected vs. non-transfected cells in two PCa cell lines (DU145 and PC3), as well as invasion in DU145 cell lines. These results indicate that miR-20a-5p expression is important for disease progression. We believe that our findings are promising for the future use of miR-20a-5p as PCa prognostic biomarker research, but further prospective validation is needed before clinical application.

## Figures and Tables

**Table 1:** miR-20a-5p expression as predictor for biochemical failure (BF), clinical failure (CF) and prostate cancer death (PCD) in prostate cancer (PCa) patients. Univariate analysis; log rank. Significant *p*-values marked in bold. Abbreviations: *n* = number; *p* = *p*-value; TS = tumor stroma; TE = tumor epithelium.

miR-20a-5p Expression		Patients		Biochemical Failure			Clinical Failure			Death of PCa		
		*n*	%	5-Year (%)	10-Year (%)	*p*	5-Year (%)	10-Year (%)	*p*	5-Year (%)	10-Year (%)	*p*
TE	Low	178	33.3	82	70	**0.001**	99	98	0.078	100	99	0.187
	High	228	42.6	69	55		95	91		99	97	
	Missing	129	24.1									
TS	Low	127	23.7	85	71	**0.003**	100	98	0.083	100	99	0.083
	High	279	52.1	70	57		96	92		99	97	
	Missing	129	24.1									
TE + TS	Low	106	19.8	86	73	**0.001**	100	98	0.051	100	99	0.216
	Mixed	93	17.4	76	64		99	98		100	99	
	High	207	38.7	68	54		95	90		99	96	
	Missing	129	24.1									

**Table 2:** Exploring miR-20a-5p impact as independent prognostic variable for BF. Multivariate analysis of miR-20a-5p expression and significant clinicopathological variables from the univariate analyses (Cox regression analyses, *n* = 535, backward conditional). Univariate analyses are in Table 1 and Supplementary Table S3. *p*-values below 0.05 marked in bold. Abbreviations: BF = biochemical failure; HR = hazard ratio; LVI = lympho-vascular infiltration; NE = not entered; NS = not significant; *p* = *p*-value; PCM = positive circumferent margin; PNI = perineural infiltration; TE = tumor epithelium; TS = tumor stroma.

Characteristic	Biochemical Failure (200 Events)			
	Model 1		Model 2	
	HR (95% CI)	*p*	HR (95% CI)	*p*
**Tumor Size**		**0.038**		**0.030**
≤20 mm	1		1	
>20 mm	1.46 (1.02–2.08)		1.48 (1.04–2.12)	
**CAPRA-S**		**<0.001**		**<0.001**
0–2	1		1	
3–5	1.60 (1.03–2.48)	**0.036**	1.60 (1.03–2.47)	**0.039**
6–12	4.26 (2.69–6.75)	**<0.001**	4.16 (2.62–6.60)	**<0.001**
**PNI**		**0.018**		**0.024**
No	1		1	
Yes	1.53 (1.07–2.17)		1.50 (1.06–2.14)	
**LVI**	NS		NS	
**PCM**	NS		NS	
**miR-20a-5p in TE**		**0.014**	NE	
Low expression	1			
High expression	1.56 (1.10–2.21)			
**miR-20a-5p in TS**	NS		NE	
**miR-20a-5p in TE + TS**	NE			**0.042**
Low/low expression			1	
Mixed expression			1.31 (0.76–2.25)	0.334
High/high expression			1.75 (1.10–2.78)	**0.018**

## Data Availability

Anonymized and limited datasets generated during and/or analyzed during the current study are available from the corresponding author on reasonable request.

## Supplementary Materials

The following are available online at https://www.mdpi.com/article/10.3390/cancers13164096/s1, Figure S1: Kaplan–Meier Survival Curves, Table S1: Wound Healing Analysis, Table S2: Invasion Analysis, Table S3: Clinicopathological Variables Predictive Value for BF and CF.
