# Supplementary material for: Overexpression of miR-20a-5p in Tumor Epithelium Is an Independent Negative Prognostic Indicator in Prostate Cancer—A Multi-Institutional Study
Source: Cancers (Basel). 2021 Aug 14;13(16):4096. doi: 10.3390/cancers13164096 (PMC8394585; doi:10.3390/cancers13164096)
Supplement: Supplementary file 1 [file cancers-13-04096-s001.zip › cancers-1318888-supplementary.pdf]

Table S1. Wound Healing Analysis.

Treatment	PC3 Absolute Migration (Average)	PC3 Graphic (C = 1)	DU145 Absolute Migration (Average)	DU145 Graphic (C = 1)
**Exp 1**				
C	47.41μm	1	146.51μm	1
miR-20a-5p	196.16μm	4.13	300.63μm	2.05
**Exp 2**				
C	69.92μm	1	88.32μm	1
miR-20a-5p	241μm	3.44	267.74μm	3.03
**Exp 3**				
C	27.83μm	1	155μm	1
miR-20a-5p	183.33μm	3.86	318.08μm	2.05

Wound Healing Analysis for PC3 and DU145 cell lines transfected with miR-20a-5p compared to controls (C=1). Both PC3 and DU145 cell line results showed, by Student t-test, significant migration ($p < 0.05$) compared to controls. Abbreviations: C = Control; Exp = experiment.

Table S2. Invasion Analysis.

Treatment	PC3 Invasion (Average)	PC3 Graphic (C=1)	DU145 Invasion (Average)	DU145 Graphic (C=1)
**Exp 1**				
C	856 cells	1	49 cells	1
miR-20a-5p	152 cells	0.17	370 cells	7.54
**Exp 2**				
C	854 cells	1	10 cells	1
miR-20a-5p	223 cells	0.26	92 cells	9.2
**Exp 3**				
C	596 cells	1	12 cells	1
miR-20a-5p	147 cells	0.24	101 cells	8.73

Invasion Analysis for PC3 and DU145 cell lines transfected with miR-20a-5p compared to controls (C=1). DU145 cell line results showed significant migration ($p < 0.05$, Student t-test) compared to controls. Abbreviations: C = Control; Exp = experiment.

Table S3. Clinicopathological variables predictive value for biochemical and clinical failure.

Characteristics	Patients *n*	Patients %	Biochemical failure (n = 200) 5-year EFS (%)	Biochemical failure *p*	Clinical failure (n = 56) 10-year EFS (%)	Clinical failure *p*
**Age**				0.237		**0.038**
≤ 65	357	67	77		94	
> 65	178	33	70		91	
**Preop PSA**				**<0.001**		**0.029**
PSA < 10	308	58	81		95	
PSA > 10	221	41	68		89	
Missing	6	1				
**ISUP Grade**				**<0.001**		**<0.001**
1	183	34	83		98	
2	219	41	77		94	
3	81	15	70		90	
4	17	3	58		86	
5	35	7	37		65	
**pT-stage**				**<0.001**		**<0.001**
pT2	374	70	83		97	
pT3a	114	21	61		87	
pT3b	47	9	43		74	

**pN-stage**				**<0.001**		**<0.001**
NX	264	49	79		96	
N0	268	50	72		90	
N1	3	1	0		33	
**Tumor size**				**<0.001**		**0.002**
0-20 mm	250	47	82		96	
≥ 20 mm	285	53	68		90	
**Free margin**				**0.049**		0.198
Yes	286	53	69		95	
No	249	47	81		90	
**PCM**				**<0.001**		**<0.001**
Yes	154	29	57		85	
No	381	71	82		96	
**PAM**				0.063		0.427
Yes	210	39	77		93	
No	325	61	74		92	
**PNI**				**<0.001**		**<0.001**
Yes	134	25	60		83	
No	401	75	80		96	
**LVI**				**<0.001**		**<0.001**
Yes	43	8	47		69	
No	492	92	77		95	
**CAPRA-S Score**				**<0.001**		**<0.001**
0-2	169	32	88		99	
3-5	258	48	78		94	
6-12	102	19	46		79	
Missing	6	1				
**Surgical proc**				0.466		0.308
Retropubic	435	81	77		92	
Perineal	100	19	67		95	

Clinicopathological variables and patient characteristics, and their predictive value for biochemical and clinical failure. (Univariate analysis; log-rank test, $n = 535$.) Significant p-values in bold. Abbreviations: EFS = event-free survival; ISUP Grade = ISUP Grade Groups; LVI = lympho-vascular infiltration; p = p-value; PAM = positive apical margin; PCM = positive circumferent margin; PNI = perineural infiltration; Preop = preoperative; PSA = prostate specific antigen; Surgical proc = Surgical procedure.

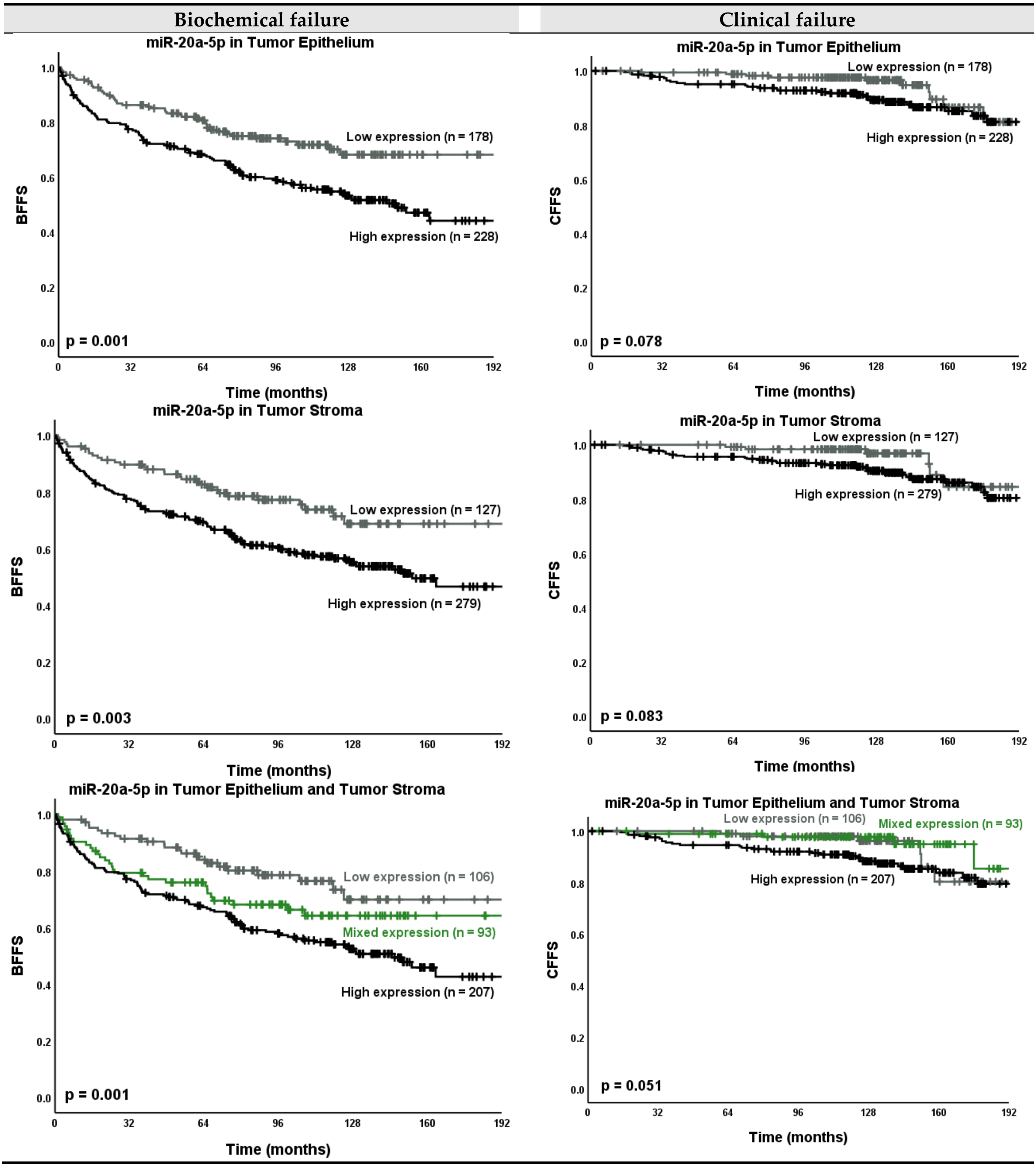

Figure S1. Kaplan-Meier survival curves presenting miR-20a-5p expression and biochemical- and clinical failure. Kaplan-Meier curves presenting the relations between low and high miR-20a-5p expression and the PCa outcomes; biochemical and clinical failure. Abbreviations: BFFS = biochemical failure-free survival; CFFS = clinical failure-free survival; *p* = p-value.
